# Preeclampsia as a Risk Factor for Diabetes: A Population-Based Cohort Study

**DOI:** 10.1371/journal.pmed.1001425

**Published:** 2013-04-16

**Authors:** Denice S. Feig, Baiju R. Shah, Lorraine L. Lipscombe, C. Fangyun Wu, Joel G. Ray, Julia Lowe, Jeremiah Hwee, Gillian L. Booth

**Affiliations:** 1 Department of Medicine, University of Toronto, Toronto, Canada; 2 Department of Health Policy, Management and Evaluation, University of Toronto, Toronto, Canada; 3 Institute for Clinical Evaluative Sciences, Toronto, Canada; 4 Department of Obstetrics & Gynecology, University of Toronto, Toronto, Canada; 5 Division of Endocrinology and Metabolism, Mount Sinai Hospital, Toronto, Canada; 6 Division of Endocrinology and Metabolism, Sunnybrook Health Sciences Centre, Toronto, Canada; 7 Women's College Research Institute and the Division of Endocrinology and Metabolism, Women's College Hospital, Toronto, Canada; 8 Keenan Research Centre, Li Ka Shing Knowledge Institute, St. Michael's Hospital, Toronto, Canada; 9 Division of Endocrinology and Metabolism, St. Michael's Hospital, Toronto, Canada; The University of Adelaide, Australia

## Abstract

**Background:**

Women with preeclampsia (PEC) and gestational hypertension (GH) exhibit insulin resistance during pregnancy, independent of obesity and glucose intolerance. Our aim was to determine whether women with PEC or GH during pregnancy have an increased risk of developing diabetes after pregnancy, and whether the presence of PEC/GH in addition to gestational diabetes (GDM) increases the risk of future (postpartum) diabetes.

**Methods and Findings:**

We performed a population-based, retrospective cohort study for 1,010,068 pregnant women who delivered in Ontario, Canada between April 1994 and March 2008. Women were categorized as having PEC alone (*n* = 22,933), GH alone (*n* = 27,605), GDM alone (*n* = 30,852), GDM+PEC (*n* = 1,476), GDM+GH (*n* = 2,100), or none of these conditions (*n* = 925,102). Our main outcome was a new diagnosis of diabetes postpartum in the following years, up until March 2011, based on new records in the Ontario Diabetes Database. The incidence rate of diabetes per 1,000 person-years was 6.47 for women with PEC and 5.26 for GH compared with 2.81 in women with neither of these conditions. In the multivariable analysis, both PEC alone (hazard ratio [HR] = 2.08; 95% CI 1.97–2.19) and GH alone (HR = 1.95; 95% CI 1.83–2.07) were risk factors for subsequent diabetes. Women with GDM alone were at elevated risk of developing diabetes postpartum (HR = 12.77; 95% CI 12.44–13.10); however, the co–presence of PEC or GH in addition to GDM further elevated this risk (HR = 15.75; 95% CI 14.52–17.07, and HR = 18.49; 95% CI 17.12–19.96, respectively). Data on obesity were not available.

**Conclusions:**

Women with PEC/GH have a 2-fold increased risk of developing diabetes when followed up to 16.5 years after pregnancy, even in the absence of GDM. The presence of PEC/GH in the setting of GDM also raised the risk of diabetes significantly beyond that seen with GDM alone. A history of PEC/GH during pregnancy should alert clinicians to the need for preventative counseling and more vigilant screening for diabetes.

*Please see later in the article for the Editors' Summary*

## Abstract 2

Denice Feig and colleagues assess the association between gestational diabetes, gestational hypertension, and preeclampsia and the development of future diabetes in a database analysis of pregnant women in Ontario, Canada.

## Editors' Summary

**Background:**

Diabetes is a chronic disease that occurs either when the pancreas does not produce enough insulin (a hormone that regulates blood sugar), known as type 1 diabetes, or when the body cannot effectively use the insulin it produces—type 2 diabetes. Raised blood sugar, is a common effect of uncontrolled diabetes and over time leads to serious complications and even death. Worryingly, the global burden of type 2 diabetes is increasing worldwide, and the World Health Organization estimates that 90% of the 347 million people with diabetes currently have type 2 diabetes. Previous studies have shown that type 2 diabetes can be prevented or delayed in high risk groups by a range of lifestyle and treatment interventions and so it is important to identify potential high risk groups to screen for type 2 diabetes.

**Why Was This Study Done?:**

Gestational diabetes (a form of diabetes that is related to pregnancy) is a major risk factor for developing type 2 diabetes. Therefore, diabetes prevention strategies should target women with gestational diabetes. Likewise, other common disorders of pregnancy possibly associated with insulin resistance, such as preeclampsia (a condition in which affected women have high blood pressure, fluid retention, and protein in their urine) and gestational hypertension (high blood pressure associated with pregnancy), may lead to the future development of type 2 diabetes. So women with these conditions may also benefit from diabetes prevention strategies. Therefore, in this large database study from Ontario, Canada, the researchers examined whether pregnant women with preeclampsia or gestational hypertension had an increased risk of developing diabetes in the years following pregnancy even if they did not have gestational diabetes.

**What Did the Researchers Do and Find?:**

The researchers used a comprehensive Canadian health database to identify all women age 15 to 50 years of age who delivered in an Ontario hospital between April 1994 and March 2008. They then identified women who had preeclampsia, gestational hypertension, or gestational diabetes through hospital records and outpatient information. The researchers then used records from the Ontario Diabetes Database to record whether these women went on to develop diabetes in the period from 180 days after delivery until March 2011.

Using these methods, the researchers identified 1,010,068 pregnant women suitable for analysis, of whom 22,933 had only preeclampsia, 27,605 had only gestational hypertension, and 30,852 had only gestational diabetes: 2,100 women had both gestational diabetes and gestational hypertension and 1,476 women had gestational diabetes and preeclampsia. Overall, 35,077 women developed diabetes (3.5%) in the follow-up period (median of 8.5 years) at a median age of 37 years. In a modeling analysis, the researchers found that women with gestational diabetes had a 15-fold increased rate of developing diabetes compared to women without gestational diabetes, gestational hypertension, and preeclampsia, while women with gestational diabetes plus either preeclampsia or gestational hypertension had a 20- to 21-fold increased rate. These results were slightly reduced after adjusting for age, income quintile, hypertension prior to pregnancy, and co-morbidity, giving a hazard ratio (HR) of 1.95 for gestational hypertension alone, an HR of 2.08 for preeclampsia alone, an HR of 12.77 for gestational diabetes alone, an HR of 18.49 for gestational diabetes plus gestational hypertension and finally, an HR of 15.75 for gestational diabetes plus preeclampsia.

**These Findings Mean?:**

These findings suggest that both preeclampsia and gestational hypertension without gestational diabetes are associated with a 2-fold increased incidence of diabetes in the years following pregnancy after controlling for several important variables. When combined with gestational diabetes, these conditions were associated with a further elevation in diabetes incidence additional to the 13-fold increased incidence resulting from gestational diabetes alone. A limitation of this study was the lack of information on obesity and body mass index, factors which are also associated with increased risk of developing diabetes. Nevertheless, these findings highlight a possible new risk factor for diabetes, and suggest that clinicians should be aware of the need for preventative measures and vigilant screening for diabetes in women with a history of preeclampsia or gestational hypertension.

**Additional Information:**

Please access these Web sites via the online version of this summary at http://dx.doi.org/10.1371/journal.pmed.1001425.

- NHS Choices has information about preeclampsia, gestational diabetes, and gestational hypertension
- Living with diabetes is a useful resource for patients with diabetes
- The Preeclampsia Foundation has more information about preeclampsia

## Introduction

The prevalence of type 2 diabetes is increasing dramatically worldwide [1] with the greatest rise in incidence occurring in adults under the age of 50, including young women [2]. Randomized trials have shown that type 2 diabetes can be prevented or delayed in high-risk groups by a variety of lifestyle and therapeutic interventions [3],[4]. However, identifying at risk populations to screen for type 2 diabetes is a critical step in translating these findings into clinical practice. Gestational diabetes is a major risk factor for the development of diabetes [5] and thus women with this condition are an ideal population to target diabetes prevention strategies. Similarly, other disorders of pregnancy associated with insulin resistance may heighten the propensity for women to develop diabetes in the years following pregnancy, and such women may also be suitable targets for diabetes prevention.

Hypertensive disorders in pregnancy, including gestational hypertension (GH) and preeclampsia (PEC), affect approximately 8% of all pregnancies [6]. More recently, women with PEC/GH have been noted to exhibit insulin resistance during pregnancy that is independent of obesity and glucose intolerance [7],[8]. Studies done during pregnancy suggest that insulin resistance predates the development of PEC, implying that insulin resistance may play a role in its etiology [7],[8]. Moreover, women with other disorders associated with insulin resistance tend to have increased rates of PEC/GH. For example, several studies have shown that women with gestational diabetes mellitus (GDM) have an increased risk of PEC/GH [9],[10], as do women with polycystic ovary syndrome [11]. The risk of PEC/GH also increases with increasing glucose intolerance [10],[12],[13].

Given the finding of insulin resistance in women with PEC/GH, and the association of PEC/GH with other disorders associated with insulin resistance in pregnancy, these conditions may be a marker of future diabetes risk, even in the absence of GDM. A few studies have looked at this association but have failed to take into account the presence of GDM. This study sought to examine whether women with PEC or GH during pregnancy have an increased risk of developing diabetes in the years following pregnancy even in the absence of GDM, and to determine whether the combination of PEC/GH and GDM confers a greater risk of postpartum diabetes, over and above the risk seen with GDM alone.

## Methods

### Ethics Statement

This protocol received ethical approval from the Institutional Review Board at Sunnybrook Health Sciences Centre in Toronto.

### Data Sources

We conducted a population-based cohort study using administrative health claims to examine the impact of PEC/GH on the subsequent risk of diabetes. The Government of Ontario acts as a single payer for all medically necessary services across a full spectrum of providers and hospitals to provide all residents of Ontario (approximately 12 million residents) with universal public health insurance. Large provincial health administrative databases are used to capture the details of this utilization and can be linked anonymously on an individual level using a unique personal identifier to provide a complete health services use profile for each resident.

### Study Population and Eligibility

We identified all women age 15 to 50 y of age who delivered in an Ontario hospital between April 1, 1994 and March 31, 2008 from the Canadian Institute for Health Information Discharge Abstract Database. For women who had multiple deliveries during the time period, one delivery was chosen at random to be the index episode from which they were followed forward in time. Women with a diagnosis of diabetes before their pregnancy were identified and excluded from the study using the Ontario Diabetes Database [14], an administrative data derived registry of Ontario residents diagnosed with non-gestational diabetes. The database has been validated against primary care charts and was shown to have a sensitivity of 86% and a specificity of greater than 97% [14]. We restricted the cohort to women who were residents of Ontario and had coverage under the province's health plan for a full 2 y prior to baseline so that we could reliably determine their diabetes status.

Women in our cohort were categorized as having PEC, GH, or GDM on the basis of their hospitalization records and outpatient data from physicians' services claims. Women who were diagnosed with both PEC and GH were assigned to the PEC group. Women assigned to the GH group had GH alone. Women whose hospitalization record at the time of delivery included a diagnostic code for diabetes or who had three or more outpatient medical claims for diabetes up to 120 d prior to their delivery were identified as having GDM. Women were considered to have a premature delivery if the delivery was <37 wk gestation.

### Baseline Variables and Study Outcomes

Women in our cohort were followed from 180 d post delivery (to avoid the potential misclassification of women with GDM undergoing routine postpartum follow-up) until March 31, 2011 for the development of diabetes, on the basis of the presence of a new record in the Ontario Diabetes Database.

We gathered demographic and clinical characteristics from administrative data to be applied in our adjusted multivariable models (see Text S1 for codes used). The age of the women was obtained through the Registered Persons Database, which contains demographic and residential information on all residents of Ontario. The Johns Hopkins Adjusted Clinical Groups (ACG) System [15] was used to create separate case-mix categories (known as collapsed ambulatory diagnostic groups [CADGs]) that reflect an individual's underlying level of morbidity. These groups were created using diagnostic codes from hospitalization records and physicians' services claims from the period 2 y prior to but excluding the delivery date. This system is a widely used method for case-mix adjustment and correlates well with health care utilization. The stable chronic medical category includes a variety of chronic conditions such as hypercholesterolemia, mitral valve disorders, atrioventricular block, and simple chronic bronchitis. The unstable chronic disease includes such conditions as ischemic heart disease, chronic obstructive lung disease, chronic liver disease, chronic kidney disease, and malignancy, among others (see Table S1 for a listing of all categories). Diagnostic codes for diabetes and hypertension were not included in the creation of the case-mix categories. Hypertension prior to pregnancy was assessed using the Ontario Hypertension Database, a validated database that identifies all individuals in Ontario who have been diagnosed with hypertension with sensitivity of 73% and a specificity of 95% [16]. For the purpose of our study, a woman was identified to have prior hypertension if the diagnosis date was greater than 280 d before the delivery. Women found to be in the Hypertension Database and diagnosed with GH were removed from the cohort, as women, by definition, cannot have GH and have hypertension prior to pregnancy. Information on individual-level income was not available in our data sources; therefore, neighborhood characteristics derived from the Canadian census were used as a proxy. The median household income level of each woman's neighborhood of residence was attributed to them using their postal code at the time of their delivery. Baseline parity was only available for a subgroup of women in our cohort who delivered between April 1, 2002 and March 31, 2008, therefore a sensitivity analysis was performed in this subset to assess the influence of parity on the association between PEC and diabetes incidence. To test whether the severity of PEC modified the association between PEC and diabetes incidence, we stratified our population of women with this condition (with or without concomitant GDM) on the basis of whether or not they had a premature delivery.

### Statistical Analysis

We calculated descriptive statistics and used one-way ANOVA, the Kruskal–Wallis test, and the chi-square test to compare the baseline characteristics of women in our different exposure groups. Incidence rates were expressed on the basis of the number of new cases of diabetes per 1,000 person-years of follow-up. A Cox proportional hazards model was used to examine the impact of PEC, GH, or GDM (with or without concomitant PEC or GH) on the risk of developing diabetes. Women were censored when follow-up was terminated; this happened at the time of death, loss of health care coverage if the women moved out of the province, if they experienced an outcome event, or at the end of the observation window. The covariates included in our model were age, socioeconomic status, hypertension (prior to pregnancy), co-morbidity, and parity (for the subset of women for whom it was available).

The proportional hazards assumption was tested [17], and interaction terms using a time-dependent covariate were inserted into the model in a second sensitivity analysis. Parity was felt to be an important covariate but was only available after 2002. For this reason a sensitivity analysis was done to assess the influence of parity on the risk of development of diabetes during the period after 2002. Women with PEC who deliver early are considered to have more severe PEC. Using PEC along with preterm delivery as a proxy for PEC severity, we performed a sub-analysis. Number needed to follow was also calculated among a random sample of 100,000 women [18]. The analyses were done using SAS version 9·2 (SAS Institute Inc).

## Results

**Figure 1:**
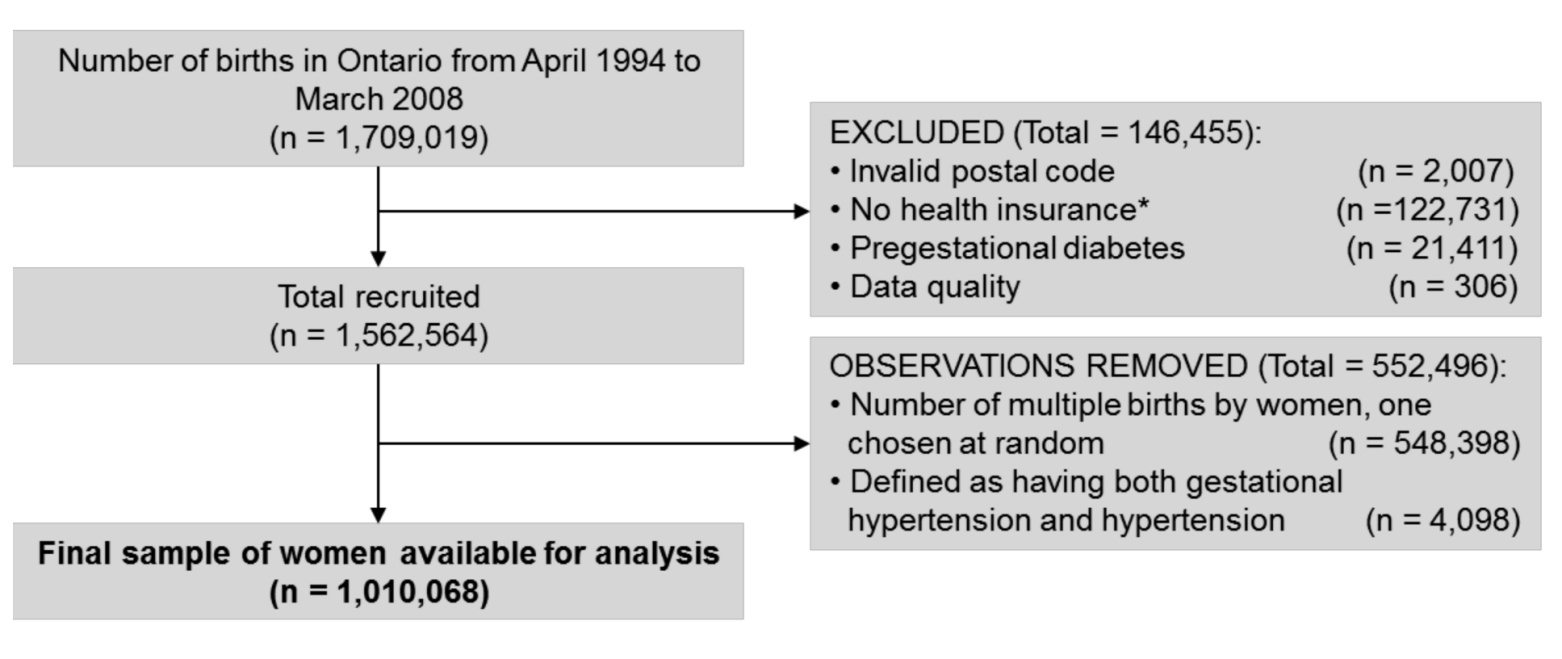
Cohort flow chart illustrating the inclusion and exclusion of participants into the study. *Mothers either lacked coverage under Ontario's Health Insurance Plan at the admission delivery date or had health insurance for less than 2 y prior to their delivery.

A total of 1,709,019 deliveries were captured in Ontario from April 1994 to March 2008. Among those that were excluded from the study, 1.3% had pregestational diabetes. A further 7.3% were excluded because they were not Ontario residents or lacked coverage under Ontario's Health Insurance Plan for a full 2 y prior to delivery (see Figure 1). Among the 1,010,068 pregnant women available for analysis, 22,933 cases of PEC alone, 27,605 cases of GH alone, 30,852 cases of GDM alone, 2,100 cases of GDM+GH, and 1,476 cases of GDM+PEC were identified. The range of follow-up was 1 d to 16.5 y, with a median follow-up of 8.5 y. Overall 35,077 women developed diabetes (3.5%).

Demographic and clinical characteristics of women included in the study, stratified by their PEC, GH, and GDM status is presented in Table 1. Women with GDM tended to be older and were more often in the lowest income quintile. Previous hypertension was more common in women with GH or PEC than women without either, particularly if they had concomitant GDM. The level of co-morbidity was low across all groups but somewhat higher in those with GDM+GH and GDM+PEC compared to women without these conditions.

**Table 1:** Demographic and clinical characteristics of women stratified by gestational diabetes diagnosis.

Characteristics	Women with No GDM			Women with GDM			*p*-Value
	*n* No GH or PEC (%)	*n* GH (%)	*n* PEC (%)	*n* GDM (%) Alone	*n* GDM+GH (%)	*n* GDM+PEC (%)	
	*n* = 925,102	*n* = 27,605	*n* = 22,933	*n* = 30,852	*n* = 2,100	*n* = 1,476	
**Age in years mean (SD)**	29.54 (5.58)	29.55 (5.76)	29.51 (5.88)	32.15 (5.17)	32.01 (5.54)	31.64 (5.73)	<0.001
**Income quintile, ** ***n*** ** (%)**							<0.001
1 (lowest)	201,597 (21.8)	5,598 (20.3)	4,901 (21.4)	8,236 (26.7)	540 (25.7)	387 (26.2)	
2	187,492 (20.3)	5,745 (20.8)	4,744 (20.7)	6,720 (21.8)	444 (21.1)	307 (20.8)	
3	188,685 (20.4)	5,811 (21.1)	4,794 (20.9)	6,366 (20.6)	413 (19.7)	322 (21.8)	
4	187,369 (20.3)	5,721 (20.7)	4,667 (20.4)	5,640 (18.3)	379 (18.0)	272 (18.4)	
5 (highest)	156,503 (16.9)	4,616 (16.7)	3,694 (16.1)	3,728 (12.1)	308 (14.7)	172 (11.7)	
Missing	3,456 (0.4)	114 (0.4)	133 (0.6)	162 (0.5)	16 (0.8)	16 (1.1)	
**Prior hypertension**	12,447 (1.3)	0 (0.0)	1,458 (6.4)	988 (3.2)	0 (0.0)	146 (9.9)	<0.001
**Co-morbidity CADG index** a							
**Chronic medical unstable**	94,212 (10.2)	3,103 (11.2)	2,854 (12.4)	5,443 (17.6)	489 (23.3)	346 (23.4)	<0.001
**Chronic medical stable**	205,115 (22.2)	7,090 (25.7)	6,221 (27.1)	9,231 (29.9)	667 (31.8)	538 (36.4)	<0.001

a Note only the two collapsed ambulatory diagnostic groups (CADGs) relating to chronic medical conditions are presented in Table 1. Nine other CADGs, relating to other categories of medical conditions, were included in the fully adjusted models. Pregnancy, however, was excluded. See Table S1.

**Figure 2:**
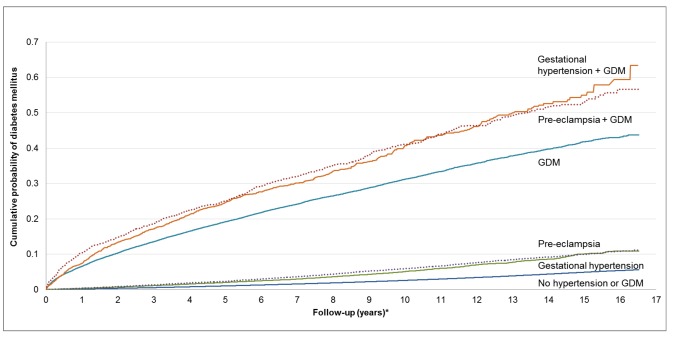
Cumulative future risk of diabetes mellitus in association with a pregnancy affected by preeclampsia, gestational hypertension, and gestational diabetes. *Follow-up period begins 180 d postpartum. This figure was produced using a competing risk method.

The unadjusted cumulative incidence of diabetes, stratified by GDM, GH, and PEC status, is presented in Figure 2. Among women in our cohort, both PEC and GH were associated with an elevated cumulative probability of developing diabetes. Women with GDM had an even higher cumulative probability of developing diabetes but this appeared to be heightened by the co-presence of PEC or GH.

The incidence rate of diabetes per 1,000 person-years is illustrated in Table 2. Women with GH or PEC had higher incidence rates of diabetes than those without either (5.26 per 1,000 person-years, 6.47 per 1,000 person-years, and 2.81 per 1,000 person-years, respectively). Those with GDM had a much higher incidence rate than those without GDM. Women with GDM and either PEC or GH had a higher incidence rate than women with GDM alone (55.02 per 1,000 person-years, 55.09 per 1,000 person-years versus 39.77 per 1,000 person-years, respectively). The mean age at diagnosis of diabetes overall was 37.34, with a median age of 37.0. The number needed to follow for 5 y to detect one case of diabetes was 4,511 for GH alone, 123 for PEC alone, 68 for GDM alone, 105 for GDM+GH, and 31 for GDM+PEC (see Table 3).

**Table 2:** Multivariable Cox proportional hazards models evaluating the relationship among preeclampsia, gestational hypertension, and gestational diabetes in the development of diabetes.

Characteristic	*n* Developed Diabetes (%)	Incidence Rate per 1,000 Person-Years	Unadjusted Analysis	Adjusted Analysis^a^
			HR (95% CI)	HR (95% CI)
No GDM, GH, or PEC	23,108 (2.5)	2.81	1 (ref)	1 (ref)
GH alone	1,085 (3.9)	5.26	1.96 (1.84–2.08)	1.95 (1.83–2.07)
PEC alone	1,510 (6.6)	6.47	2.25 (2.14–2.37)	2.08 (1.97–2.19)
GDM alone	8,082 (26.2)	39.77	14.83 (14.46–15.22)	12.77 (12.44–13.10)
GDM+GH	681 (32.4)	55.09	21.06 (19.51–22.73)	18.49 (17.12–19·.96)
GDM+PEC	611 (41.4)	55.02	19.80 (18.27–21.45)	15.75 (14.52–17.07)

a Adjusted for age, income quintile, prior hypertension, and co–morbidity using John Hopkins collapsed ambulatory diagnostic groups (CADG).

**Table 3:** Number needed to follow.

Follow-up Period	Number Needed to Follow, *n*				
	GH	PEC	GDM	GDM+GH	GDM+PEC
2 y	13,109	263	150	241	67
5 y	4,511	123	68	105	31
8.5 y (median)	2,332	83	44	66	21
16.5 (maximum)	1,152	60	29	41	15

In the unadjusted Cox proportional hazards model, women who had GH or PEC during pregnancy had double the risk of developing diabetes in the 16.5 y following delivery, compared to women who had neither during pregnancy (Table 2). Women with GDM had a 15-fold increased rate of developing diabetes over those without GDM, PEC, or GH, while women with GDM+PEC and GDM+GH during pregnancy had the highest rates with a 20- to 21-fold increased rate. These effects were only modestly reduced after adjusting for age, income quintile, hypertension prior to pregnancy, and co-morbidity, (hazard ratio [HR] 1.95 [1.83–2.07] for GH alone, HR 2.08 [1.97–2.19] for PEC alone, HR 12.77 [12.44–13.10] for GDM alone, HR 18.49 [17.12–19.96] for GDM+GH, HR 15.75 [14.52–17.07] for GDM+PEC) (Table 2). See Table S2 for the number of women at risk at the end of each follow-up year.

A sensitivity analysis was done to assess the influence of parity on the risk of development of diabetes using data from 2002–2008. The association between future DM and GH/PEC and GDM became even stronger when adjusting for parity (along with age, income quintile, prior hypertension, and co-morbidity) (HR for GH alone 1.82 [95% CI 1.63–2.03], HR for PEC alone 1.92 [95% CI 1.63–2.28], HR for GDM alone 16.56 [95% CI 15·81–17.34], HR for GDM+GH 23.48 [21.01–26.24], HR for GDM+PEC 22.53 [18.54–27.38]).

In the second sensitivity analysis, a time-dependent covariate was added into the adjusted model as an interaction term. There was a time-varying effect among the groups on the risk of developing diabetes, especially those with GDM. The risk for developing diabetes in women with GH and PEC stayed fairly stable at the 2-y, and median follow-up times (GH HR = 1.99 for all times; PEC HR = 2.26 and HR = 2.01, respectively). For those with GDM, however, the risk of developing diabetes decreased over time compared with the women without GH/PEC or GDM (HR = 19.09 at 2 y, HR = 8.20, at median follow-up of 8.5 y). Similar patterns were seen in the GDM+GH and the GDM+PEC groups (HR = 25.28, HR = 12.86; and HR = 24.54, HR = 11.13, at 2 and 8.5 y, respectively).

**Figure 3:**
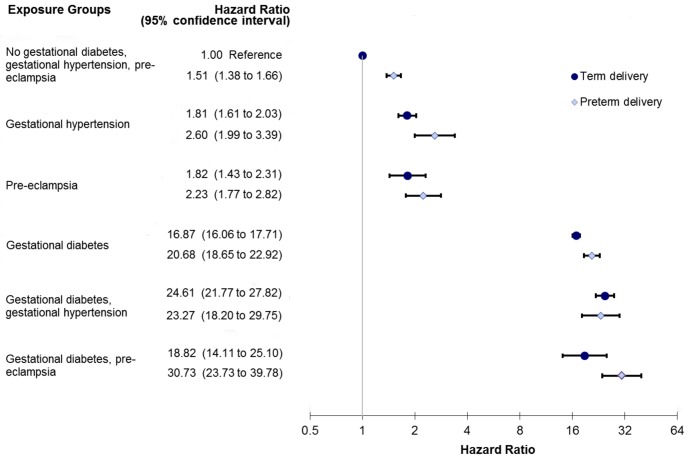
Sub-analysis modeling the relationship among preeclampsia, gestational hypertension, gestational diabetes, and preterm delivery in the development of diabetes. Model also adjusts for age, income quintile, prior hypertension, and co-morbidity using the Johns Hopkins collapsed ambulatory diagnostic group (CADG). The bands represent 95% confidence intervals.

A sub-analysis was performed looking at PEC severity by assessing PEC along with preterm delivery. There was a trend towards a greater incidence of diabetes among women with PEC who had a preterm delivery (Figure 3). Those women with GDM and PEC and preterm delivery experienced the highest risk of developing diabetes, HR 30.73 (95% CI 23.73–39.78) (Figure 3).

## Discussion

In this study the presence of PEC or GH, in the absence of GDM, was associated with a 2-fold increased incidence of diabetes when followed up to 16.5 years after pregnancy, after controlling for several important confounding variables. In the setting of GDM, these conditions were associated with a further elevation in diabetes risk, over and above the already substantial (∼13-fold) increase resulting from GDM alone. This risk was even higher in the setting of preterm delivery, suggesting that diabetes incidence rises with increasing severity of these hypertensive disorders. These findings highlight a possible new risk factor for diabetes, and support the need to counsel patients with hypertensive disorders of pregnancy regarding postpartum diabetes screening prevention.

Common pathogenic pathways may underlie the association between both GDM and PEC/GH leading to an increased risk of diabetes. Firstly, each of these conditions is associated with insulin resistance [7],[8],[11],[19],[20]. Not only do women with PEC/GH have insulin resistance during pregnancy, several studies have also found higher levels of insulin resistance in women with a history of PEC/GH years after delivery, even after controlling for body mass index and excluding women with previous GDM [21],[22]. Women with a history of PEC/GH also show manifestations of the metabolic syndrome years after delivery, a syndrome known for its association with insulin resistance [23]–[26]. Endothelial dysfunction and markers of chronic vascular inflammation have been found in women with PEC as well as women with GDM, both during and after pregnancy [27]–[30]. These entities have been shown to precede the development of overt hyperglycemia in patients at risk for type 2 diabetes [30]. We hypothesize that these common mediators are likely to increase the risk of type 2 diabetes in women with a history of PEC.

We found that the risk of developing diabetes over the years was stable for those with GH and PEC alone; however, the risk decreased over time in women with GDM. It may be that those with a very high risk develop diabetes early on. Those who remain have an inherently lower risk leading to the lower HR ratio observed. This finding is consistent with what we know about the natural history of diabetes postpartum. Some groups develop diabetes at a very high rate early on (50% within 5 y of delivery) [31].

Three previous studies looked at the risk of developing type 2 diabetes in women with a history of PEC/GH. In one study, women enrolled in the Mater–University of Queensland Study of Pregnancy between 1981 and 1984 who had PEC/GH at baseline were 1.76 times more likely to report having developing diabetes 21 y later [32]. However, approximately one-half of their original cohort were lost to follow-up. In a Danish cohort of women with PEC or GH, the risk of diabetes postpartum was also found to be increased over a median of 14.6 years [33]. Neither of these preceding studies were able to isolate the risk imparted by PEC/GH from that of GDM. A third registry study of women with PEC in Norway also found an increased risk of diabetes in women with PEC, however follow-up was short, only 3.7 y, and the diagnosis of diabetes was made in women using medications for diabetes, possibly under-estimating the true incidence of diabetes [34].

Strengths of our study include that this study was a large, population-based study with long and complete follow-up (over 1,000,000 women followed for up to 16.5 y). Our study also examined the risk of diabetes associated with both GH and PEC, while controlling for the presence of GDM, and examined the combined impact of GDM and PEC/GH together. We were also able to examine the severity of PEC with the addition of preterm delivery. Some limitations of this study include our inability to adjust for clinical factors, in particular, obesity, which in itself is associated with insulin resistance, and is a well-known risk factor for the development of GDM [35] and PEC [36]. Obesity is not well captured in our inpatient hospitalization database, nor is it well coded in our outpatient database, which is based on physician outpatient billing codes. Furthermore, family history, physical activity, glucose, and blood pressure measures are also known risk factors. We were, however, able to adjust for several other important confounding variables including maternal age, prior hypertension, income quintile, parity, and co-morbidity. One previous study was able to adjust for obesity and physical activity, and the risk of developing diabetes was still significant [30]. From our data sources we could not differentiate type 1 from type 2 diabetes; however, given the mean age at diagnosis was 37.4 y, it is most likely that the majority of the women developed type 2 diabetes in the years after pregnancy. Women with a history of GDM may be offered routine glucose testing postpartum, thus leading to more diagnoses of diabetes compared with women with a history of PEC. This situation may have led to under-reporting of diabetes in women with a history of PEC; therefore the rate of development of diabetes in these women may even be higher than reported. Follow-up of women with hypertensive disease in pregnancy may lead to more interactions with the health care system compared to women without hypertensive diseases in pregnancy, thus leading to more diabetes testing and diagnosis and a possible bias towards overestimating the risk; however, there is currently no standard recommendation to screen women with GH/PEC for diabetes postpartum. Our capture of GH and PEC may have been incomplete. In a study looking at the accuracy of hospital data in the perinatal period using ICD-10 codes, when diagnosing GH and PEC, sensitivity was reduced (58.6% and 50.0%, respectively), however specificity was excellent (99% and 99.8%, respectively) [37]. Finally, the algorithms used to describe a number of the variables used in the study (GDM, PEC, GH) have not been formally validated.

In summary, in this large, population-based study, we found that the presence of either PEC or GH during pregnancy were strong predictors for the development of diabetes years following the pregnancy, and the presence of PEC or GH in a woman with a history of GDM increased the risk of diabetes over and above that observed with GDM alone. These findings have important implications for maternal health, especially given the increase in obesity-related diseases. A history of PEC or GH during pregnancy should alert clinicians to the need for preventative counseling and more vigilant screening for diabetes.

## Supporting Information

Table S1 Distribution of co-morbidity categories (collapsed ambulatory diagnostic groups)* among women stratified by gestational diabetes diagnosis. *Collapsed ambulatory diagnostic groups [CADGs] were created using the Johns Hopkins Adjusted Clinical Groups (ACG) System. CADG category number 12 (pregnancy) was not included in the analyses.
(DOCX)

Table S2 Number of women at risk at the end of each follow-up year in Ontario, from 1994 to 2011. *Year 0 indicates the beginning of the follow-up period.
(DOCX)

Text S1 Codes for variables used.
(DOCX)

## Abbreviations

GDM: gestational diabetes
GH: gestational hypertension
HR: hazard ratio
PEC: preeclampsia

## References

[1] Zimmet PZ, Alberti KGMM (2006) Introduction: globalization and the non–communicable disease epidemic. Obesity 14: 1–3. 16493116 10.1038/oby.2006.1

[2] Lipscombe LL, Hux JE (2007) Trends in diabetes prevalence, incidence, and mortality in Ontario, Canada 1995–2005: a population-based study. Lancet 369: 750–756. 17336651 10.1016/S0140-6736(07)60361-4

[3] Diabetes Prevention Program Research Group (2002) N Eng J Med 346: 393–403.

[4] Tuomilehto J, Lindström J, Eriksson JG, Valle TT, Hämäläinen H, et al (2001) Prevention of type 2 diabetes mellitus by changes in lifestyle among subjects with impaired glucose tolerance. N Eng J Med 344: 1343–1350. 10.1056/NEJM200105033441801 11333990

[5] Feig DS, Zinman B, Wang X, Hux JE (2008) Risk of development of diabetes mellitus after diagnosis of gestational diabetes. CMAJ 179: 229–234. 18663202 10.1503/cmaj.080012 PMC2474881

[6] National High Blood Pressure Education Program Working Group (2000) Report of the National High Blood Pressure Education Program Working Group on high blood pressure in pregnancy. Am J Obstet Gynecol 183: S1–S22. 10920346

[7] Parretti E, lapolla A, Dalfra MG, Pacini G, Mari A, et al (2006) Preeclampsia in lean normotensive normotolerant pregnant women can be predicted by simple insulin sensitivity indexes. Hypertension 47: 449–453. 16446386 10.1161/01.HYP.0000205122.47333.7f

[8] Sierra–Laguado J, Garcia RG, Celedón J, Arenas-Mantilla M, Pradilla LP, et al (2007) Determination of insulin resistance using the homeostatic model assessment (HOMA) and its relation with the risk of developing pregnancy–induced hypertension. Am J Hypertens 20: 437–442. 17386353 10.1016/j.amjhyper.2006.10.009

[9] Bryson CL, Ioannou GN, Rulyak SJ, Critchlow C (2003) Association between gestational diabetes and gestational hypertension. Am J Epidemiol 158: 1148–1153. 14652299 10.1093/aje/kwg273

[10] Vambergue A, Nuttens MC, Goeusse P, Biausque S, Lepeut M, et al (2002) Pregnancy induced hypertension in women with gestational carbohydrate intolerance: the diagest study. European J Obs Gyne Repro Bio 102: 31–35. 10.1016/s0301-2115(01)00556-5 12039086

[11] Legro RS (2009) Insulin resistance in women's health: why it matters and how to identify it. Curr Opin Obstet Gynecol 21: 301–305. 19550327 10.1097/GCO.0b013e32832e07d5 PMC3590839

[12] Joffe GM, Esterlitz JR, Levine RJ, Clemens JD, Ewell MG, et al (1998) The relationship between abnormal glucose tolerance and hypertensive disorders of pregnancy in healthy nulliparous women. Calcium for Preeclampsia Prevention (CPEP) Study Group. Am J Obstet Gynecol 179: 1032–1037. 9790393 10.1016/s0002-9378(98)70210-8

[13] Sermer M, Naylor CD, Gare DJ (1995) Impact of increasing carbohydrate intolerance on maternal–fetal outcomes in 3637 women without gestational diabetes. Am J Obstet Gynecol 173: 146–156. 7631672 10.1016/0002-9378(95)90183-3

[14] Hux JE, Ivis F, Flintoft V, Bica A (2002) Diabetes in Ontario: determination of prevalence and incidence using a validated administrative data algorithm. Diabetes Care 25: 512–516. 11874939 10.2337/diacare.25.3.512

[15] Johns Hopkins University (2011) The Johns Hopkins ACG® Case-Mix System Applications Guide Version 10.0 December 2011. Available: http://www.acg.jhsph.org. Accessed 31 January 2013.

[16] Tu K, Campbell NR, Chen Z, Cauch–Dudek K, McAlister FA (2007) Accuracy of administrative databases in identifying patients with hypertension. Open Medicine 1: 18–26. PMC2801913 20101286

[17] Schoenfeld D (1980) Chi-squared goodness-of-fit tests for the proportional hazards regression model. Bometrika 67: 145–153.

[18] Austin PC (2010) Absolute risk reductions and numbers needed to treat can be obtained from adjusted survival models for time-to-event outcomes. J Clin Epi 63: 46–55. 10.1016/j.jclinepi.2009.03.012 19595575

[19] Ryan EA, Imes S, Liu D, McManus R, Finegood DT, et al (1995) Defects in insulin secretion and action in women with a history of gestational diabetes. Diabetes 44: 506–512. 7729607 10.2337/diab.44.5.506

[20] D'Anna R, Baviera G, Corrado F, Giordano D, De Vivo A, et al (2006) Adiponectin and insulin resistance in early– and late–onset preeclampsia. BJOG 113: 1264–1269. 17010118 10.1111/j.1471-0528.2006.01078.x

[21] Fuh MMT, Yin CS, Pei D, Sheu WH, Jeng CY, et al (1995) Resistance to insulin–mediated glucose uptake and hyperinsulinemia in women who had preeclampsia during pregnancy. Am J Hypertens 8: 768–771. 7546505 10.1016/0895-7061(95)00078-4

[22] Soonthornpun K, Soonthornpun S, Wannaro P, Setasuban W, Thamprasit A (2009) Insulin resistance in women with a history of severe pre–eclampsia. J Obstet Gynaecol Res 35: 55–59. 19215548 10.1111/j.1447-0756.2008.00865.x

[23] Ray JG (2004) Dysmetabolic syndrome, placenta-mediated disease and future risk of cardiovascular disease. Fetal and Mat Med Rev 15: 231–246.

[24] Sattar N, Ramsay J, Crawford L, Cheyne H, Greer IA (2003) Classic and novel risk factor parameters in women with a history of preeclampsia. Hypertension 12: 12. 10.1161/01.HYP.0000074428.11168.EE 12743016

[25] Girouard J, Giguere Y, Moutquin J-M, Forest J-C (2007) Previous hypertensive disease of pregnancy is associated with alterations of markers of insulin resistance. Hypertension 49: 1056–1062. 17389257 10.1161/HYPERTENSIONAHA.107.087528

[26] Smith GN, Walker MC, Liu A, Wen SW, Swansburg M, et al (2009) A history of preeclampsia identifies women who have underlying cardiovascular risk factors. Am J Obstet Gynecol 200: 58.e1–58.e8. 18691690 10.1016/j.ajog.2008.06.035

[27] Agatisa PK, Ness RB, Roberts JM, Costantino JP, Kuller LH, et al (2004) Impairment of endothelial function in women with a history of preeclampsia: an indicator of cardiovascular risk. Am J Phsiol Circ Physiol 286: H1389–H1393. 10.1152/ajpheart.00298.2003 15020302

[28] Kvehaugen AS, Dechend R, Ramstad HB, Troisi R, Fugelseth D, et al (2011) Endothelial function and circulating biomarkers are disturbed in women and children after preeclampsia. Hypertension 58: 63–69. 21606387 10.1161/HYPERTENSIONAHA.111.172387

[29] Hawfield A, Freedman BI (2009) Pre–eclampsia: the pivotal role of the placenta in its pathophysiology and markers for early detection. Ther Adv Cardiovasc Dis 3: 65–73. 19124387 10.1177/1753944708097114 PMC2752365

[30] Caballero AE (2004) Endothelial dysfunction, inflammation, and insulin resistance: a focus on subjects at risk for type 2 diabetes. Curr Diab Rep 4: 237–246. 15265464 10.1007/s11892-004-0074-9

[31] Kjos SL, Peters RK, Xiang A, Henry OA, Montoro M, et al (1995) Predicting future diabetes in Latino women with gestational diabetes. Utility of early postpartum glucose tolerance testing. Diabetes 44: 586–591. 7729620 10.2337/diab.44.5.586

[32] Callaway LK, Lawlor DA, O'Callaghan M, Williams GM, Najman JM, et al (2007) Diabetes mellitus in the 21 years after a pregnancy that was complicated by hypertension: findings from a prospective cohort study. Am J Obstet Gynecol 197: 492.e1–492.e7. 17980185 10.1016/j.ajog.2007.03.033

[33] Lykke JA, Langhoff-Roos J, Sibai BM, Funai EF, Triche EW, et al (2009) Hypertensive pregnancy disorders and subsequent cardiovascular morbidity and type 2 diabetes mellitus in the mother. Hypertension 53: 944–951. 19433776 10.1161/HYPERTENSIONAHA.109.130765

[34] Engeland A, Bjorge T, Kjersti Daltveit AK, Skurtveit S, Vangen S, et al (2011) Risk of diabetes after gestational diabetes and preelampsia. A registry-based study of 230,000 women in Norway. Eur J Epidemiol 26: 157–163. 21298469 10.1007/s10654-010-9527-4 PMC3043244

[35] Singh J, Huang C-C, Driggers RW, Timofeev J, Amini D, et al (2012) The impact of pre-pregnancy body mass index on the risk of gestational diabetes. J Maternal Fetal Neonatal Med 25: 5–10. 10.3109/14767058.2012.626920 21955004

[36] O'Brien TE, Ray JG, Chan W-S (2003) Maternal body mass index and the risk of preeclampsia: a systematic overview. Epi 14: 368–374. 10.1097/00001648-200305000-00020 12859040

[37] Taylor LK, Travis S, Pym M, Olive E, Henderson-Smart DJ (2005) How useful are hospital morbidity data for monitoring conditions occurring in the perinatal period? Austr NZ J Obs Gyn 45: 36–41. 10.1111/j.1479-828X.2005.00339.x 15730363

