# Supplementary material for: Preeclampsia as a Risk Factor for Diabetes: A Population-Based Cohort Study
Source: PLoS Med. 2013 Apr 16;10(4):e1001425. doi: 10.1371/journal.pmed.1001425 (PMC3627640; doi:10.1371/journal.pmed.1001425)
Supplement: Table S1 — Distribution of co-morbidity categories (collapsed ambulatory diagnostic groups)* among women stratified by gestational diabetes diagnosis. *Collapsed ambulatory diagnostic groups [CADGs] were created using the Johns Hopkins Adjusted Clinical Groups (ACG) System. CADG category number 12 (pregnancy) was not included in the analyses. (DOCX) [file pmed.1001425.s001.docx]

Table S1: Distribution of comorbidity categories (Collapsed Ambulatory Diagnostic Groups)* among women stratified by gestational diabetes diagnosis

|  | **Women with no gestational diabetes** | | | **Women with gestational diabetes** | | | p-value |
| --- | --- | --- | --- | --- | --- | --- | --- |
|  | No gestational hypertension or pre-eclampsia | Gestational hypertension | Pre-eclampsia | Gestational diabetes alone | Gestational diabetes + gestational hypertension | Gestational diabetes + pre-eclampsia |  |
|  | N=925,102 | N=27,605 | N=22,933 | N=30,852 | N=2,100 | N=1,476 |  |
| CADG* |  |  |  |  |  |  |  |
| 1. Acute Minor | 774,138 (83.7%) | 22,757 (82.4%) | 19,495 (85.0%) | 26,549 (86.1%) | 1,782 (84.9%) | 1,260 (85.4%) | <0.001 |
| 2. Acute Major | 771,188 (83.4%) | 22,946 (83.1%) | 19,546 (85.2%) | 27,392 (88.8%) | 1,808 (86.1%) | 1,304 (88.3%) | <0.001 |
| 3. Likely to Recur | 629,127 (68.0%) | 18,148 (65.7%) | 15,843 (69.1%) | 21,933 (71.1%) | 1,414 (67.3%) | 1,058 (71.7%) | <0.001 |
| 4. Asthma | 64,571 (7.0%) | 2,308  (8.4%) | 1,971 (8.6%) | 2,313 (7.5%) | 196  (9.3%) | 159 (10.8%) | <0.001 |
| 5. Chronic Medical: Unstable* | 94,212 (10.2%) | 3,103 (11.2%) | 2,854 (12.4%) | 5,443 (17.6%) | 489  (23.3%) | 346 (23.4%) | <0.001 |
| 6. Chronic Medical: Stable* | 205,115 (22.2%) | 7,090 (25.7%) | 6,221 (27.1%) | 9,231 (29.9%) | 667  (31.8%) | 538 (36.4%) | <0.001 |
| 7. Chronic Specialty: Stable | 17,620 (1.9%) | 593  (2.1%) | 535 (2.3%) | 573  (1.9%) | 58  (2.8%) | 37  (2.5%) | <0.001 |
| 8. Eye/Dental | 61,471 (6.6%) | 1,637  (5.9%) | 1,697 (7.4%) | 2,181 (7.1%) | 134  (6.4%) | 140  (9.5%) | <0.001 |
| 9. Chronic Specialty: Unstable | 28,299 (3.1%) | 907  (3.3%) | 768 (3.3%) | 1,151 (3.7%) | 76  (3.6%) | 60  (4.1%) | <0.001 |
| 10. Psychosocial | 337,843 (36.5%) | 10,060 (36.4%) | 8,841 (38.6%) | 11,620 (37.7%) | 833  (39.7%) | 605 (41.0%) | <0.001 |
| 11. Preventive/Administrative | 549,123 (59.4%) | 15,575 (56.4%) | 13,050 (56.9%) | 17,472 (56.6%) | 1,111 (52.9%) | 772 (52.3%) | <0.001 |

CADG = Collapsed Ambulatory Diagnostic Groups

*CADGs were created using the Johns Hopkins Adjusted Clinical Groups (ACG) System.

CADG category #12 (Pregnancy) was not included in the analyses..
