# Supplementary material for: Preeclampsia as a Risk Factor for Diabetes: A Population-Based Cohort Study
Source: PLoS Med. 2013 Apr 16;10(4):e1001425. doi: 10.1371/journal.pmed.1001425 (PMC3627640; doi:10.1371/journal.pmed.1001425)
Supplement: Table S2 — Number of women at risk at the end of each follow-up year in Ontario, from 1994 to 2011. *Year 0 indicates the beginning of the follow-up period. (DOCX) [file pmed.1001425.s002.docx]

Table S2 Number of women at risk at the end of each follow-up year in Ontario, from 1994 to 2011

| Year | **Women with no gestational diabetes** | | | **Women with gestational diabetes** | | | Total |
| --- | --- | --- | --- | --- | --- | --- | --- |
|  | No gestational hypertension or pre-eclampsia | Gestational hypertension | Pre-eclampsia | Gestational diabetes alone | Gestational diabetes + gestational hypertension | Gestational diabetes + pre-eclampsia |  |
| 0* | 925,102 | 27,605 | 22,933 | 30,852 | 2,100 | 1,476 | 1,010,068 |
| 1 | 923,178 | 27,495 | 22,831 | 28,817 | 1,944 | 1,324 | 1,005,589 |
| 2 | 913,892 | 27,182 | 22,598 | 27,388 | 1,804 | 1,252 | 994,116 |
| 3 | 865,034 | 25,035 | 21,814 | 24,549 | 1,581 | 1,154 | 939,167 |
| 4 | 777,526 | 21,473 | 20,436 | 20,542 | 1,294 | 1,041 | 842,312 |
| 5 | 698,698 | 18,247 | 19,288 | 17,247 | 1,051 | 943 | 755,474 |
| 6 | 627,856 | 15,404 | 18,140 | 14,408 | 863 | 846 | 677,517 |
| 7 | 561,937 | 12,846 | 17,085 | 12,093 | 694 | 770 | 605,425 |
| 8 | 499,357 | 10,490 | 16,095 | 10,177 | 548 | 688 | 537,355 |
| 9 | 438,962 | 8,605 | 14,581 | 8,354 | 431 | 595 | 471,528 |
| 10 | 378,354 | 7,178 | 12,660 | 6,710 | 330 | 476 | 405,708 |
| 11 | 317,557 | 5,733 | 10,587 | 5,487 | 258 | 393 | 340,015 |
| 12 | 263,402 | 4,501 | 8,817 | 4,400 | 192 | 316 | 281,628 |
| 13 | 215,512 | 3,578 | 7,134 | 3,486 | 153 | 254 | 230,117 |
| 14 | 162,823 | 2,662 | 5,312 | 2,456 | 107 | 189 | 173,549 |
| 15 | 102,916 | 1,618 | 3,375 | 1,501 | 61 | 110 | 109,581 |
| 16 | 36,260 | 540 | 1,195 | 516 | 22 | 39 | 38,572 |

*Year 0 indicates the beginning of the follow-up period.
