# Supplementary material for: Preeclampsia as a Risk Factor for Diabetes: A Population-Based Cohort Study
Source: PLoS Med. 2013 Apr 16;10(4):e1001425. doi: 10.1371/journal.pmed.1001425 (PMC3627640; doi:10.1371/journal.pmed.1001425)
Supplement: Text S1 — Codes for variables used. (DOCX) [file pmed.1001425.s003.docx]

TextS1 Codes for Variables Used

Preeclampsia

For admissions up to 280 days prior to delivery, any of the following codes were identified as an indicator of PET:

Using CIHI-DAD + SDS to Identify:

Prior to 2002

Any diagnosis field:

dxcode in 64240-64244, 64250-64254, 64260 – 64264, 64270-64274

2002 onwards

Any diagnosis field:

dx10code in, O14, O15

Gestational Hypertension

For admissions up to 280 days prior to delivery, any of the following codes were identified as an indicator of GH:

Using CIHI-DAD + SDS to Identify:

Prior to 2002

Any diagnosis field:

dxcode in 64200-64204, 64230 – 64234,

64290-64294,

2002 onwards

Any diagnosis field:

dx10code in, O13, O16

Gestational Diabetes

Identify any of the following codes were used as an indicator of GDM:

i) Not in ODD (Ontario Diabetes Database) >= 280 days before delivery date

AND

iia) Index hospitalization (delivery hospitalization) record with any diagnosis code: dx9code in (‘250’ ‘648.0’ ‘648.8’) or dx10code in (‘E10, E11, E13, E14’ or ‘O24’)

OR

iib) In the 280 days prior to index hospitalization 3 OHIP codes of 250. These must be non-lab OHIP claims. Multiple claims on the same day should not be counted separately.

All records without these codes: GDM = 0

if i AND (iia or iib) then gdm = 1
